# Unraveling population trends in Italy (1921–2021) with spatial econometrics

**DOI:** 10.1038/s41598-023-46906-2

**Published:** 2023-11-21

**Authors:** Leonardo Salvatore Alaimo, Clio Ciaschini, Francesca Mariani, Eva Cudlinova, Michele Postigliola, Donatella Strangio, Luca Salvati

**Affiliations:** 1 Department of Social Sciences and Economics, Sapienza University of Rome, Piazzale Aldo Moro 5, 00185 Rome, Italy; 2 Department of Social and Economic Sciences, Polytechnic University of Marche, Piazzale C. Martelli 8, 60121 Ancona, Italy; 3 Department of Economics, University of South Bohemia, Branišovská 1645/31A, České Budějovice 2, 370 05 Ceske Budejovice, Czech Republic; 4 Department of Methods and Models for Economics, Territory and Finance, Faculty of Economics, Sapienza University of Rome, Via del Castro Laurenziano 9, 00161 Rome, Italy

**Keywords:** Environmental economics, Socioeconomic scenarios, Sustainability

## Abstract

Testing density-dependence and path-dependence in long-term population dynamics under differentiated local contexts contributes to delineate the changing role of socioeconomic forces at the base of regional disparities. Despite a millenary settlement history, such issue has been rarely investigated in Europe, and especially in highly divided countries such as those in the Mediterranean region. Using econometric modeling to manage spatial heterogeneity, our study verifies the role of selected drivers of population growth at ten times between 1921 and 2021 in more than 8000 Italian municipalities verifying density-dependent and path-dependent dynamics. Results of global and quantile (spatial) regressions highlight a differential impact of density and (lagged) population growth on demographic dynamics along the urban cycle in Italy. Being weakly significant in the inter-war period (1921–1951), econometric models totalized a high goodness-of-fit in correspondence with compact urbanization (1951–1981). Model’s fit declined in the following decades (1981–2021) reflecting suburbanization and counter-urbanization. Density-dependence and path-dependence were found significant and, respectively, positive or negative, with compact urbanization, and much less intense with suburbanization and counter-urbanization. A spatial econometric investigation of density-dependent and path-dependent mechanisms of population dynamics provided an original explanation of metropolitan cycles, delineating the evolution of socioeconomic (local) systems along the urban-rural gradient.

## Introduction

Urban cycles have been extensively studied all over the world, and especially in advanced economies, since centuries^1–3^. Being regarded as separate–and recurrent–stages of a long-term cycle, urbanisation (mostly driven by positive natural balances of population and internal migration) was found associated with settlement concentration and economic agglomeration—possibly stimulating residential mobility to outer areas as a response to congestion externalities^4–6^. The subsequent suburbanization wave has in turn affected metropolitan structures and socioeconomic functions, determining a (more or less intense) decline of central cities^7–9^. Short-haul mobility, preference for specific dwellings in rural locations and sudden changes in local job markets driven by technology and accessibility gains fueled a later counter-urbanisation^10–12^. A renewed impulse to centralised urban growth to catch the intrinsic benefits of scale economies finally characterised recent population trends in advanced countries (the so-called ’re-urbanisation’ wave).

Assuming demographic dynamics as one of the most relevant processes at the base of urban cycles, the analysis of local-scale population trends may clarify the recent evolution of cities and the emergence (or consolidation) of spatial disparities across regions and countries^13–20^. In Europe, a wealth of factors has reported to affect the spatial distribution of resident population^21–24^, including globalization, structural change of economic systems, and international migration^25–28^. Such trends - especially in Mediterranean Europe - have reflected intense economic downturns leading to urbanisation-reurbanisation sequences accelerated by a rapid demographic transition toward low fertility, higher life expectancy, and rising immigration^6,11,29^.

In this context, self-regulation of population growth is a candidate driver of demographic dynamics at regional scale^30–32^. Empirical studies have demonstrated the existence of a density-dependent regulation in human populations^33,34^. However, density-dependent mechanisms can explain only a part of the overall variability of demographic growth rates along urban-rural gradients^35^, being in turn mediated by individual choices/preferences, cultural, ethnic and religious factors, and exogenous processes of a stochastic nature. These factors were not always identifiable and easily modelled^36–38^, being associated sometimes with the notion of ‘path dependency’. Similarly to density-dependence, path-dependent regulation of population growth and decline exerted a variable impact on demographic dynamics depending on the local context, e.g. in correspondence with other socioeconomic processes that accelerate or limit its action^39–42^.

Temporal volatility^43^, spatial heterogeneity^44^, and the intrinsic stochasticity^45^ underlying the complex mosaic of growth and decline typical of human populations, all observed under demographic conditions of dynamic equilibrium^46–48^, were the characteristics of density-dependent and path-dependent mechanisms at the base of population dynamics along sufficiently long time ranges and over geographical areas large enough to be representative of short and medium-range mobility. Despite the intrinsic importance of these issues for both research and policy, empirical analyses decomposing path-dependent socioeconomic transformations from density-dependent mechanisms of population growth (and decline) were rather scarce in advanced economies^42,49,50^. A comparative investigation of the (positive or negative) feedback mechanisms at the base of population dynamics appears indispensable for regional science and applied economics, considering together density-dependence and path-dependence, and controlling for the role of space^51–53^ over time intervals long enough to investigate the intrinsic impact of distinctive background contexts^15,50,54^. In this perspective, the sequential stages of urban cycles are the appropriate background influencing density-dependent and path-dependent mechanisms of population growth and decline^55^. In other words, we assume that the individual stages of the cycle (namely, urbanisation, suburbanisation, counter-urbanisation, and re-urbanisation) may differently shape population dynamics, because of the diverging impact of density-dependent and path-dependent regulation along the urban-rural gradient^56–58^.

Sharing comparable demographic outcomes at the regional scale and over long time periods^59^, Mediterranean countries represent appropriate cases when defining internal and external factors that may influence local-scale population dynamics^60–62^. In this perspective, density-dependent and path-dependent regulation of population dynamics were tested at the municipal scale in Italy over a sufficiently long and homogeneous time interval (1921–2021) representative of sequential stages of a building cycle from urbanisation to re-urbanisation^14,63,64^. We adopted an econometric specification that quantifies the impact of density-dependent and path-dependent processes on population dynamics^65^, controlling for economic (agglomeration, scale, accessibility, amenities), and non-economic (e.g. spatial) effects^42,66,67^. A comparative scrutiny of models’ results will contribute regional science with a more complete understanding of metropolitan cycles - an issue of vital importance when assessing urban-rural relationships that evolve over time.

## Data and methods

### Study area

The investigated area includes Italy (301,330 km$^2$) geographically partitioned into three macro-regions (North, Centre, South) and 20 administrative regions^68^. Italy shows evident disparities in Northern and Southern Italy as far as economic growth, social development, and land resources are concerned^15,69^, with the latter region classified as marginal and disadvantaged^70–72^. As in other Mediterranean countries, the urban-rural divide in Italy is also particularly accentuated, delineating different socioeconomic contexts from large (and mostly mono-centric) metropolitan areas (Rome, Milan, Naples, Turin) to hyper-rural areas along the Apennine mountain chain, mainly in Southern Italy. Italy shows extensive socioeconomic disparities between Northern and Southern regions. Taken together, these features make Italy a paradigmatic case allowing a refined investigation of the interplay of environmental and socioeconomic dimensions at the base of urban cycles in Southern Europe.

### Data and variables

As a basic element of European classification of territorial units, Local Administrative Units (LAU) have a key role in official statistics because of data availability from national censuses and relevance for implementation of local policy^73^. Since LAUs were subjected to minor changes over long observation times, Istat disseminated a homogenized list of spatial units and boundaries for cross-region and cross-country comparisons^50^. Estimates of resident population at this spatial level were made available at the municipal scale approximately every 10 years over a century. Homogenized census data were derived from Istat (1994) and updated from the warehouse released by the Italian National Statistical Institute. The most recent data (2021) were derived from the national population register (the base of the ‘permanent census’ progressively replacing the traditional population censuses in Italy since 2018), whose results were aligned with the 2011 (and earlier) census(es). Population size and density, as well as the annual growth rate, were the main variables in our study. Population growth rates (%) were calculated at each municipality over 10 time intervals of similar length (1921–1931, 1931–1936, 1936–1951, 1951–1961, 1961–1971, 1971–1981, 1981–1991, 1991–2001, 2001–2011, and 2011–2021). Population density (inhabitants/km2) and population size (absolute number of resident inhabitants) were calculated at the same spatial scale for 11 time points between 1921 and 2021 and expressed as logarithms^63^. Lagged population growth rates and population density allow an explicit test of, respectively, path-dependence and density-dependence^65^. Population size was used to test the importance of agglomeration, as a measure of urban concentration^8^.

### Econometric analysis

We assumed different spatial regimes of population dynamics associated with each stage of the cycle^64^, modelling the variability in population growth rates ($Pop.Growth_{(t=2,1)}$) as a function of (i) population growth rate ($Pop.Growth_{(t=2,1)}$) in the previous (i.e. $lag_{(-1)}$) time interval, (ii) population density ($Dem.Density_{(t=0)}$), and (iii) the overall size of the resident population as a proxy of agglomeration ($Pop.Size_{(t=0)}$), both measured at the beginning of the related observation time. The analysis has also considered (iv) an average measure of *Elevation* for each municipality, as derived from the official source of Istat municipal atlas, (v) a dummy of closeness to the sea coastline (*Sea*.*Prox*) classifying each municipality as ‘coastal’ (code 1) or ‘inland’ (code 0) and (vi) a dummy separating municipalities acting as the ‘head town’ (*Cap*.*city*) of a given province (with code 1) from the remaining municipalities classified with code 0. All variables were standardised prior to analysis^50^. Use of these variables in econometric models testing density-dependent and path-dependent mechanisms of population dynamics was discussed in^42,50,63^. Model specification was summarised as follows:

1 $$\begin{aligned} \begin{aligned} Pop.Growth_{(t=2,1)}&= \alpha + \beta _1 Pop.Growth_{(t=1,0)} + \beta _2 Dem.Density_{(t=0)} + \beta _3 Pop.Size_{(t=0)} + \beta _4 Elevation + \\&\quad + \beta _5 Sea.Prox + \beta _6 Cap.city + \epsilon \end{aligned} \end{aligned}$$

where $\alpha$ is the regression constant (model’s intercept), $\beta _1, \beta _2. \dots , \beta _6$ are the regression coefficients (slope), and $\epsilon$ is the stochastic error of the model. Models were run controlling for time (i.e. distinguishing the impact of the four stages of the cycle mentioned above) and space (i.e. using spatial econometrics approaches whose results were compared with those from standard approaches). The adopted specification allow (i) discriminating the impact of economic from non-economic forces of population growth, (ii) distinguishing the role of density-dependent mechanisms of population growth and decline from the more general path-dependency of local population dynamics, (iii) highlighting the importance of direct spatial effects, and (iv) separating them from the indirect ones (i.e. spillovers). The individual stages of the metropolitan cycle in Italy were defined as follows^63^: urbanisation (1951–1981), suburbanisation (1981–2001), counter-urbanisation mixed with early re-urbanisation (2001–2021); population dynamics during inter-war decades were more mixed and prepared the system to the sudden shift toward compact urbanisation. Cross-section regressions were run assuming each observation decade as a separate stage of the cycle representative of specific socioeconomic contexts and population dynamics at the local scale. A Variance Inflation Factor (VIF) was finally calculated for each time interval. Values systematically below 5 for all variables delineate a non-redundant structure of predictors’ matrix, in line with the basic assumption of non-collinearity typical of most econometric models.

#### Standard models

Assuming linear changes over time in population distribution over space, Eq. (1) was preliminary tested with a linear specification adopting global Ordinary Least Squares (OLS) regressions. The models’ goodness of fit was checked by way of adjusted $R^2$ coefficients and the Akaike Information Criterion (AIC). Inference on regression results (i.e. Fisher-Snedecor *F* tests and Student *t* tests respectively on the overall regression fit and on individual coefficients, testing against the null hypothesis of zero coefficients with $p < 0.001$) provided an additional criterion for model’s evaluation^74^. To verify the violations of the basic assumptions of a general linear model, a Durbin-Watson (*DW*) statistic checking for serial correlation, a Breusch-Pagan (*BP*) index for heteroscedasticity, and a Moran (*M*) spatial autocorrelation coefficient for spatial dependence of residuals were run for each model, testing for significance at $p < 0.05$ against the null hypothesis of no serial correlation, no heteroscedasticity, and no spatial autocorrelation structure, respectively.

#### Spatially explicit models

**Figure 1:**
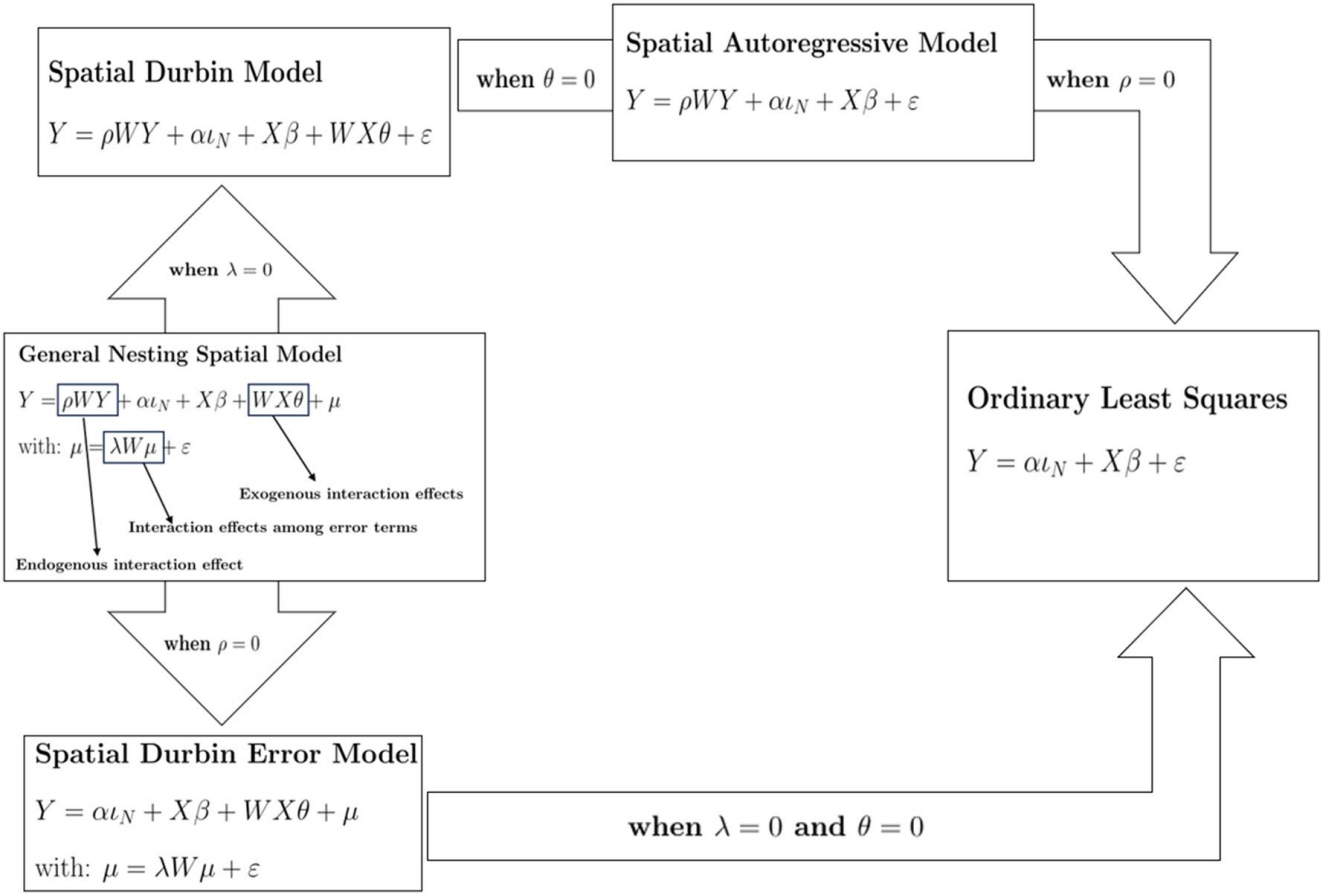
Graphical description of the spatial models adopted in the work.

Equation (1) was additionally estimated comparing the results of global models that make spatial relations explicit using spatial weights among municipalities calculated as (i) a contiguity ($0-1$) Queen matrix (*Q*) and (ii) a linear distance matrix (*W*). While presenting a variable goodness-of-fit, consistent regression outputs (i.e. the same significant predictors with comparable intensity and sign) may identify a statistically stable (and conceptually relevant) relationship between population growth rates and the selected predictors^42^. By investigating the dependence of a given variable’s values on the values of the same variable recorded at neighbouring locations, spatial autocorrelation assumes outcome in one area to be affected by outcomes, covariates or errors in nearby areas, meaning that models may contain spatial lags of the outcome variable, spatial lag of covariates, and autoregressive errors, respectively^56^. Regressions run in this study include a Spatial Autoregressive Model (SAR), a spatial autoregressive error term (SDE), and a Spatial Durbin Model (SDM). A flowchart of the adopted spatial models has been shown in Figure 1.

Both direct and indirect (spillover) effects between municipalities were detected. Best-fit estimation of the proposed models using empirical data was evaluated using pseudo $R^2$. As in the case above, non-parametric (quantile) regressions with spatial weights separately from *Q* and *W* matrices were run to estimate Eq. 1 at four percentiles of the dependent variable assuming (i) deviation from normality, (ii) non-linear dependence between predictors and the dependent variable, and (iii) spatial relations among input variables. Model’s outcomes include estimates of intercept and slope coefficients and the associated significance level testing for the null-hypothesis of non-significant regression coefficient at $p < 0.05$.

## Results

### The interwar period

Ordinary Least Square (OLS) regressions performed on standardized input variables show positive and significant values of lagged population growth rates and proximity to the sea coast despite a relatively small adjusted-$R^2$ (0.05) typical of the time period (1921–1951) preceding the sharp wave of urbanisation in the aftermath of World War II. The regression coefficient for head towns is also positive. Negative and significant coefficients are observed for elevation. The variance inflation factor (VIF) is systematically low and largely below 5, suggesting a non-redundant structure of the predictor matrix (Table 1). The results obtained from quantile regressions confirm the results of the OLS regression with reference to lagged population growth, population size, elevation, proximity to the sea coast and head towns. In contrast, the differences with the OLS regression emerge when referring to population density. In general, the slope coefficients of lagged population growth rates increase from the first ($\tau = 0.25$) to the fourth ($\tau = 0.99$) quartile. Starting from the coefficients of the variable linked to population density, the sign–in the first three quartiles—is consistent with the one performed in the OLS, while in the fourth quartile is negative. A significant and positive coefficient is also shown by the variable linked to the size of the population from the first to the third quartile, to then become negative–even if significant–if referred to the fourth quartile. With reference to the last two variables, i.e. proximity to the sea and head town, the coefficient maintains the sign of OLS model with highly significant values in the quartiles from $\tau = 0.25$ to $\tau = 0.75$ for the first variable, and no significance in $\tau = 0.99$ for the first predictor. With reference to the second predictor, no coefficient appears significant except that of the third and forth quartiles, maintaining a coherent coefficient sign with OLS model. Compared to OLS models, lower AIC values have been shown in the first three quartiles of the standard quantile regression; a higher value is shown when $\tau =0.99$.

**Table 1:** Results of standard (OLS, Ordinary Least Square, and quantile) regressions as well as global (SAR: Spatial Autoregressive model; SDE: Spatial Error model; SDM: Spatial Durbin model) and quantile spatial models run with both contiguity and linear distance spatial weighting matrices; population growth rate (% annual) in 1931–1936 as dependent variable; population growth rate (1921–1931), demographic density (1921), population size (1921), elevation, proximity to the sea coast and a dummy indicating municipalities that act as provincial head town as predictors (*$p< 0.05$; **$0.001<$ $p< 0.05$; ***$p< 0.001$).

Predictor	OLS	Quantile regression				VIF	
		$\tau = 0.25$	$\tau = 0.50$	$\tau = 0.75$	$\tau = 0.99$		
Intercept	$\approx 0.000$	-0.192	0.005	0.189	1.485		
	(0.010)	(0.005)***	(0.003)	(0.004)***	(0.836)*		
Pop.growth	0.079	0.350	0.401	0.388	0.347	1.02	
	(0.011)***	(0.031)***	(0.031)***	(0.036)***	(0.392)		
Dem.density	− 0.129	− 0.011	− 0.028	− 0.039	− 0.120	1.32	
	(0.012)***	(0.003)***	(0.004)***	(0.004)***	(0.055)**		
Pop.size	− 0.004	0.046	0.033	0.015	− 0.107	1.38	
	(0.012)	(0.004)***	(0.004)***	(0.005)	(0.048)**		
Elevation	− 0.142	− 0.045	− 0.016	− 0.005	− 0.042	1.40	
	(0.012)***	(0.003)***	(0.004)***	(0.005)	(0.075)		
Sea prox.	0.045	0.028	0.036	0.043	0.106	1.18	
	(0.011)***	(0.003)***	(0.004)***	(0.004)***	(0.115)		
Cap.city	0.091	− 0.001	0.006	0.021	5.387	1.13	
	(0.011)***	(0.003)	(0.004)	(0.004)***	(0.654)		
Lag. Pop.growth							
Lag.Dem.density							
Lag.Pop.size							
Lag.Elevation							
Lag.Sea prox.							
Lag.Cap.city							
Breusch-Pagan	5204.2***						
Durbin-Watson	1.89**						
Slope equality			10.8***				
Moran’s I(z)							
W spatial matrix							
Adjusted-$R^2$	0.047	0.098	0.104	0.100	0.088		
AIC	22,604	8,684	6,722	8,425	33,576		

Predictor	Contiguity-based spatial weights						
	SAR	SDE	SDM	Quantile regression			
				$\tau = 0.25$	$\tau = 0.50$	$\tau = 0.75$	$\tau = 0.99$
Intercept	$\approx 0.000$	$\approx 0.000$	$\approx 0.000$	− 0.176	0.006	0.152	4.710
	(0.010)	(0.011)	(0.010)	(0.015)***	(0.004)	(0.007)***	(0.657)***
Pop.growth	0.078	0.077	0.075	0.344	0.380	0.350	0.178
	(0.010)***	(0.010)***	(0.010)***	(0.079)***	(0.043)***	(0.028)***	(0.227)
Dem.density	− 0.127	− 0.130	− 0.132	− 0.010	− 0.020	− 0.032	− 0.048
	(0.012)***	(0.012)***	(0.013)***	(0.006)	(0.005)***	(0.006)***	(0.519)
Pop.size	− 0.007	− 0.008	− 0.020	0.042	0.027	$\approx 0.000$	− 1.666
	(0.012)	(0.012)	(0.013)	(0.006)***	(0.006)***	(0.006)	(0.528)**
Elevation	− 0.143	− 0.151	− 0.200	− 0.046	− 0.019	− 0.015	− 0.691
	(0.012)***	(0.013)***	(0.014)***	(0.006)***	(0.005)***	(0.004)***	(0.770)
Sea prox.	0.042	0.038	0.002	0.026	0.028	0.033	0.121
	(0.011)***	(0.011)***	(0.012)	(0.006)***	(0.006)***	(0.005)***	(1.067)
Cap.city	0.092	0.093	0.094	0.000	0.008	0.024	0.648
	(0.011)***	(0.011)***	(0.011)***	(0.004)	(0.004)**	(0.006)***	(0.246)
Lag. Pop.growth			0.012				
			(0.010)				
Lag.Dem.density			0.039				
			(0.023)*				
Lag.Pop.size			0.080				
			(0.023)***				
Lag.Elevation			0.184				
			(0.023)***				
Lag.Sea prox.			0.124				
			(0.021)***				
Lag.Cap.city			− 0.055				
			(0.026)**				
Breusch-Pagan							
Durbin-Watson							
Slope equality							
Moran’s I(z)				0.0304***			
W spatial matrix				ns	*	***	ns
Adjusted-$R^2$	0.050	0.051	0.060				
AIC	22,592	22,533	22,533				

Predictor	Distance-based spatial weights						
	SAR	SDE	SDM	Quantile regression			
				$\tau = 0.25$	$\tau = 0.50$	$\tau = 0.75$	$\tau = 0.99$
Intercept	$\approx 0.000$	− 0.039	− 0.008	− 0.133	0.006	0.152	1.417
	(0.010)	(0.107)	(0.011)	(0.004)***	(0.003)*	(0.004)***	(0.288)***
Pop.growth	0.066	0.065	0.067	0.216	0.288	0.319	0.366
	(0.010)***	(0.010)***	(0.010)***	(0.080)***	(0.050)***	(0.024)***	(0.102)***
Dem.density	− 0.103	− 0.136	− 0.136	0.016	0.007	− 0.015	− 0.112
	(0.012)***	(0.013)***	(0.013)***	(0.005)***	(0.004)	(0.005)***	(0.041)***
Pop.size	− 0.043	− 0.042	− 0.040	0.012	− 0.011	− 0.029	− 0.097
	(0.012)***	(0.014)***	(0.014)***	(0.005)	(0.005)***	(0.006)***	(0.040)**
Elevation	− 0.161	− 0.213	− 0.221	− 0.064	− 0.042	− 0.041	− 0.046
	(0.013)***	(0.014)***	(0.014)***	(0.006)***	(0.005)***	(0.005)***	(0.065)
Sea prox.	0.015	− 0.003	− 0.004	− 0.005	0.012	0.019	0.105
	(0.011)	(0.012)	(0.012)	(0.004)***	(0.003)***	(0.005)***	(0.103)
Cap.city	0.099	0.101	0.100	0.007	0.016	0.03	5.385
	(0.011)***	(0.011)***	(0.011)***	(0.004)**	(0.003)***	(0.004)***	(2.654)**
Lag. Pop.growth			0.765				
			(0.196)***				
Lag.Dem.density			0.236				
			(0.062)***				
Lag.Pop.size			0.122				
			(0.087)				
Lag.Elevation			0.433				
			(0.052)***				
Lag.Sea prox.			− 0.055				
			(0.044)				
Lag.Cap.city			0.040				
			(0.588)				
Breusch-Pagan							
Durbin-Watson							
Slope equality							
Moran’s I(z)				0.0282***			
W spatial matrix				***	***	**	*
Adjusted-$R^2$	0.070	0.077	0.078				
AIC	22,422	22,360	22,352				

The results of the econometric tests indicate OLS estimations as partly biased, since tests for serial correlation, heteroscedasticity, and spatial dependence are all significant. Thus, we adopted spatial econometric techniques starting from a Spatial Durbin model (hence SDM) for the global regressions by explicitly considering the spatial structure of the input data and we compared the results with reference techniques such as Spatially Autoregressive (SAR), and Spatial Error (SDE) models. In direct and indirect SDM regressions we find positive coefficients related to the variable describing lagged population growth rates, coherently with the OLS regression. This predictor displays significant and positive coefficients for both direct and indirect effects. With reference to population size and density, elevation, as well as head town, we observe opposite signs considering direct and indirect effects. For instance, the indirect effect of population size is negative and statistically significant, while being positive but statistically insignificant when considering direct effects. Proximity to the sea coast has a positive impact (both direct and indirect) on population growth rates. Moving on to spatially quantile regressions, significant and negative coefficients were found for elevation - even if the intensity of this significance is strongest in the first three quartiles and then decreases in the fourth quartile. Positive coefficients, on average, are associated with lagged population growth and demographic density, proximity to the sea coast, and head town. Finally, the positive role of head town on population growth is positive and significant at least in the second, third and forth quartile. Positive coefficients are associated with population size in the first and second quartile, reverting to a negative coefficient in the forth quartile. Similar results were found comparing the outcomes of quantile regressions run with spatial matrices based on contiguity and linear distances among municipalities. Models based on the distance-weighted matrix tend to have lower AIC values than models based on contiguity. These results are in line with $R^2$, which, in this case, shows greater values than contiguity-based spatial models. In particular, SDM performs the lowest value of AIC whereas SAR performs the greatest value of AIC in distance weighted models. When considering contiguity, SDE and SDM produce the lowest AIC values, while SAR performs the greatest AIC value. Generally speaking, the goodness of fit of spatial models is consistently higher than that of standard models.

### The breakdown of intense urbanisation

Moving to the subsequent time interval, Table 2 illustrates the results of standard econometrics and spatial models using both contiguity and linear distance spatial weights. Here the results of OLS estimates and standard quantile regressions appear rather coherent in assigning positive and significant coefficients to lagged population growth rates, demographic density, proximity to the sea coast and head town, with increasing values of the adjusted-$R^2$. Econometric diagnostics also indicate that the OLS estimate is (moderately) biased. The tests of serial correlation, heteroscedasticity and spatial dependence are all significant, suggesting the appropriateness of using spatial models. When comparing the econometric results from different spatial weighting schemes, models such as SAR and SDE give similar results with OLS as far as sign and significance of the regression coefficients. A general comparison of OLS values of AIC with that of spatial weighted models highlight that SAR and SDM seem to be more effective than OLS in explaining the variance of the phenomenon, while SDE has an higher value of AIC in comparison to OLS. These results are also consistent with the estimate of direct impacts from SDM. Indirect impacts from SDM are rather different from the structure of direct impacts, suggesting the role of spatial heterogeneity for specific predictors, such as demographic density, population size, and elevation. In general, regressions based on a spatially weighted distance matrix seem to perform better than those based on contiguity. With distance, the three AIC values are consistent with the corresponding $R^2$ (lower in SAR and greater in SDM). With contiguity, SDM displays a lowest AIC value that corresponds to the greater $R^2$ value. Considering quantile regressions, lagged population growth, elevation, and proximity to the sea coast maintain the OLS coefficients’ sign and significance almost for all quartiles. Population size coefficients were found positive for the first three quartiles, becoming negative in the fourth quartile.

Considering a subsequent time interval with intense expansion of human settlements in Italy, lagged population growth rates, demographic density, elevation, proximity to the sea coast, and head town display almost positive and significant regression coefficients both for OLS and quantile regressions, with markedly improved $R^2$ (Table 3). The positive coefficients estimated for demographic density and population size are antithetical to those observed for earlier time windows. Variance inflation factors (VIFs) for each predictor are systematically less than 5, suggesting a non-redundant structure of the regressors matrix. Test diagnostics for serial correlation, heteroscedasticity, and spatial dependence suggest the appropriateness of using spatial modelling. Considering both contiguity and distance weighting, SAR and SDE results are rather consistent with those of OLS with respect to the sign of the regression coefficients. The direct effects of SDM mostly resemble the outcome of SAR and SDE models. Direct and indirect coefficients for demographic density and population size assume opposite signs. Spatial models, in general, have higher values of AIC than OLS. Quantile regressions provided similar outcomes irrespective of the spatial weighting scheme adopted.

**Table 2:** Results of standard (OLS, Ordinary Least Square, and quantile) regressions as well as global (SAR: Spatial Autoregressive model; SDE: Spatial Error model; SDM: Spatial Durbin model) and quantile spatial models run with both contiguity and linear distance spatial weighting matrices; population growth rate (% annual) in 1936–1951 as dependent variable; population growth rate (1931–1936), demographic density (1931), population size (1931), elevation, proximity to the sea coast and a dummy indicating municipalities that act as provincial head town as predictors (* $p< 0.05$; ** $0.001<$ $p< 0.05$; *** $p< 0.001$).

Predictor	OLS	Quantile regression				VIF	
		$\tau = 0.25$	$\tau = 0.50$	$\tau = 0.75$	$\tau = 0.99$		
Intercept	$\approx 0.000$	− 0.455	− 0.064	0.385	1.960		
	(0.010)	(0.009)***	(0.008)***	(0.010)***	(0.098)***		
Pop.growth	0.171	0.460	0.466	0.466	0.462	1.03	
	(0.010)***	(0.035)***	(0.023)***	(0.021)***	(0.318)		
Dem.density	0.012	0.114	0.100	0.062	− 0.107	1.39	
	(0.012)	(0.010)***	(0.009)***	(0.011)***	(0.080)		
Pop.size	0.091	0.141	0.063	0.008	− 0.201	1.43	
	(0.012)***	(0.008)***	(0.009)***	(0.011)	(0.076)***		
Elevation	− 0.094	− 0.007	− 0.017	− 0.047	− 0.375	1.44	
	(0.012)***	(0.010)	(0.009)*	(0.013)***	(0.086)***		
Sea prox.	0.121	0.058	0.080	0.098	0.243	1.18	
	(0.011)***	(0.009)***	(0.010)***	(0.010)***	(0.178)		
Cap.city	0.024	$\approx 0.000$	0.018	0.046	0.144	1.14	
	(0.011)*	(0.006)	(0.011)	(0.010)***	(0.035)***		
Lag. Pop.growth							
Lag.Dem.density							
Lag.Pop.size							
Lag.Elevation							
Lag.Sea prox.							
Lag.Cap.city							
Breusch-Pagan	74.5						
Durbin-Watson	1.60 ***						
Slope equality			32.6***				
Moran’s I(z)							
W spatial matrix							
Adjusted-$R^2$	0.096	0.109	0.088	0.080	0.282		
AIC	22,176	17,456	17,269	19,527	36,820		

Predictor	Contiguity-based spatial weights						
	SAR	SDE	SDM	Quantile regression			
				$\tau = 0.25$	$\tau = 0.50$	$\tau = 0.75$	$\tau = 0.99$
Intercept	$\approx 0.000$	$\approx 0.000$	$\approx 0.000$	− 0.400	− 0.060	0.315	1.524
	(0.010)	(0.013)	(0.010)	(0.013)***	(0.009)***	(0.018)***	(0.237)***
Pop.growth	0.162	0.158	0.156	0.425	0.425	0.406	0.421
	(0.010)***	(0.010)***	(0.010)***	(0.131)***	(0.068)***	(0.048)***	(0.131)***
Dem.density	0.008	− 0.002	− 0.012	0.095	0.092	0.055	− 0.121
	(0.012)	(0.012)	(0.013)	(0.015)***	(0.011)***	(0.012)***	(0.049)**
Pop.size	0.082	0.095	0.094	0.138	0.067	0.001	− 0.183
	(0.012)	(0.012)***	(0.013)	(0.010)***	(0.010)***	(0.011)	(0.070)***
Elevation	− 0.098	− 0.114	− 0.141	− 0.016	− 0.025	− 0.061	− 0.412
	(0.012)***	(0.013)***	(0.014)***	(0.010)	(0.010)	(0.014)	(0.079)***
Sea prox.	0.107	0.104	0.086	0.053	0.068	0.085	0.139
	(0.011)***	(0.011)***	(0.022)***	(0.011)***	(0.009)***	(0.013)***	(0.354)
Cap.city	0.027	0.024	0.027	0.002	0.019	0.044	0.156
	(0.011)**	(0.011)**	(0.011)**	(0.009)	(0.009)**	(0.011)***	(0.032)***
Lag. Pop.growth			0.064 (0.024)***				
Lag.Dem.density			0.086 (0.022)***				
Lag.Pop.size			− 0.063 (0.023)***				
Lag.Elevation			0.123 (0.022)***				
Lag.Sea prox.			0.072 (0.021)***				
Lag.Cap.city			− 0.003 (0.025)				
Breusch-Pagan							
Durbin-Watson							
Slope equality							
Moran’s I(z)				0.127***			
W spatial matrix				***	***	***	*
Adjusted-$R^2$	0.132	0.132	0.137				
AIC	21,941	24,094	21,901				

Predictor	Distance-based spatial weights						
	SAR	SDE	SDM	Quantile regression			
				$\tau = 0.25$	$\tau = 0.50$	$\tau = 0.75$	$\tau = 0.99$
Intercept	0.007	0.205	− 0.010	− 0.376	− 0.069	0.297	1.886
	(0.010)	(0.492)	(0.010)	(0.007)***	(0.008)***	(0.010)***	(0.112)***
Pop.growth	0.145	0.142	0.141	0.265	0.342	0.325	0.458
	(0.010)***	(0.010)***	(0.010)***	(0.109)**	(0.051)***	(0.041)***	(0.148)***
Dem.density	0.067	0.066	0.064	0.096	0.122	0.121	− 0.095
	(0.011)***	(0.013)***	(0.013)***	(0.012)***	(0.013)***	(0.014)***	(0.073)
Pop.size	0.043	0.080	0.086	0.132	0.053	− 0.006	− 0.200
	(0.012)***	(0.013)***	(0.013)***	(0.010)***	(0.011)***	(0.010)	(0.083)**
Elevation	− 0.095	− 0.120	− 0.139	− 0.042	− 0.033	− 0.059	− 0.378
	(0.012)***	(0.014)***	(0.014)***	(0.010)***	(0.009)***	(0.015)***	(0.089)***
Sea prox.	0.079	0.089	0.085	0.031	0.042	0.065	0.231
	(0.011)***	(0.012)***	(0.012)***	(0.007)***	(0.006)***	(0.012)***	(0.190)
Cap.city	0.037	0.026	0.021	0.015	0.020	0.038	0.146
	(0.010)***	(0.010)**	(0.010)**	(0.006)**	(0.008)**	(0.012)***	(0.031)***
Lag. Pop.growth			− 0.350 (0.119)**				
Lag.Dem.density			− 0.390 (0.051)***				
Lag.Pop.size			0.419 (0.086)***				
Lag.Elevation			0.345 (0.043)***				
Lag.Sea prox.			− 0.265 (0.040)***				
Lag.Cap.city			− 3.436 (0.550)***				
Breusch-Pagan							
Durbin-Watson							
Slope equality							
Moran’s I(z)				0.097***			
W spatial matrix				***	***	***	*
Adjusted-$R^2$	0.179	0.190	0.199				
AIC	21,424	23,353	21,240

**Table 3:** Results of standard (OLS, Ordinary Least Square, and quantile) regressions as well as global (SAR: Spatial Autoregressive model; SDE: Spatial Error model; SDM: Spatial Durbin model) and quantile spatial models run with both contiguity and linear distance spatial weighting matrices; population growth rate (% annual) in 1951–1961 as dependent variable; population growth rate (1936–1951), demographic density (1936), population size (1936), elevation, proximity to the sea coast and a dummy indicating municipalities that act as provincial head town as predictors (*$p< 0.05$; **$0.001<$ $p< 0.05$; ***$p< 0.001$).

Predictor	OLS	Quantile regression				VIF	
		$\tau = 0.25$	$\tau = 0.50$	$\tau = 0.75$	$\tau = 0.99$		
Intercept	$\approx 0.000$	-0.427	-0.047	0.339	2.163	1.07	
	(0.009)	(0.009)***	(0.007)***	(0.009)***	(0.141)***		
Pop.growth	0.378	0.491	0.579	0.664	1.184		
	(0.010)***	(0.015)***	(0.013)***	(0.016)***	(0.194)***		
Dem.density	0.202	0.167	0.159	0.155	0.387	1.41	
	(0.011)***	(0.011)***	(0.008)***	(0.011)***	(0.120)***		
Pop.size	− 0.035	$\approx 0.000$	− 0.036	− 0.833	− 0.312	1.47	
	(0.011)**	(0.010)	(0.008)***	(0.010)***	(0.107)***		
Elevation	0.029	0.086	0.055	0.029	0.063	1.45	
	(0.011)*	(0.010)***	(0.009)***	(0.010)***	(0.114)		
Sea prox.	0.101	0.067	0.062	0.075	0.198	1.20	
	(0.010)***	(0.009)***	(0.007)***	(0.010)***	(0.186)		
Cap.city	0.068	0.076	0.064	0.070	0.002	1.14	
	(0.010)***	(0.008)***	(0.007)***	(0.005)***	(0.061)		
Lag. Pop.growth							
Lag.Dem.density							
Lag.Pop.size							
Lag.Elevation							
Lag.Sea prox.							
Lag.Cap.city							
Breusch-Pagan	1760.0***						
Durbin-Watson	1.64***						
Slope equality			24.9***				
Moran’s I(z)							
W spatial matrix							
Adjusted-$R^2$	0.225	0.178	0.220	0.239	0.227		
AIC	20,928	17,225	16,649	18,979	38,6670		

Predictor	Contiguity-based spatial weights						
	SAR	SDE	SDM	Quantile regression			
				$\tau = 0.25$	$\tau = 0.50$	$\tau = 0.75$	$\tau = 0.99$
Intercept	$\approx 0.000$	$\approx 0.000$	$\approx 0.000$	− 0.410	− 0.047	0.348	2.104
	(0.009)	(0.012)	(0.009)	(0.012)***	(0.006)***	(0.012)***	(0.263)***
Pop.growth	0.362	0.361	0.354	0.478	0.577	0.669	1.168
	(0.010)***	(0.010)***	(0.010)***	(0.028)***	(0.017)***	(0.016)***	(0.197)***
Dem.density	0.192	0.196	0.180	0.163	0.157	0.159	0.400
	(0.011)***	(0.011)***	(0.012)***	(0.014)***	(0.010)***	(0.012)***	(0.089)***
Pop.size	− 0.024	− 0.007	0.019	0.006	− 0.036	− 0.085	− 0.286
	(0.011)**	(0.012)	(0.012)	(0.011)	(0.009)***	(0.009)***	(0.104)***
Elevation	0.024	0.022	0.004	0.085	0.055	0.033	0.093
	(0.011)**	(0.012)*	(0.013)	(0.012)***	(0.010)***	(0.016)**	(0.155)
Sea prox.	0.098	0.107	0.111	0.065	0.062	0.076	0.227
	(0.010)***	(0.010)***	(0.011)***	(0.011)***	(0.007)***	(0.011)***	(0.159)
Cap.city	0.068	0.062	0.058	0.075	0.065	0.069	− 0.018
	(0.010)***	(0.010)***	(0.010)***	(0.008)***	(0.008)***	(0.007)***	(0.046)
Lag. Pop.growth			0.080				
			(0.020)***				
Lag.Dem.density			− 0.013				
			(0.021)				
Lag.Pop.size			− 0.191				
			(0.021)***				
Lag.Elevation			0.041				
			(0.021)*				
Lag.Sea prox.			− 0.060				
			(0.019)***				
Lag.Cap.city			0.043				
			(0.023)*				
Breusch-Pagan							
Durbin-Watson							
Slope equality							
Moran’s I(z)				0.109***			
W spatial matrix				ns	ns	ns	ns
Adjusted-$R^2$	0.242	0.244	0.257				
AIC	20,800	20,669	20,674				

Predictor	Distance-based spatial weights						
	SAR	SDE	SDM	Quantile regression			
				$\tau = 0.25$	$\tau = 0.50$	$\tau = 0.75$	$\tau = 0.99$
Intercept	0.007	0.202	0.005	− 0.355	− 0.498	0.304	2.319
	(0.009)	(0.325)	(0.010)	(0.008)***	(0.008)***	(0.009)***	(0.173)***
Pop.growth	0.334	0.346	0.337	0.424	0.514	0.618	1.247
	(0.010)***	(0.010)***	(0.010)***	(0.020)***	(0.018)***	(0.015)***	(0.161)***
Dem.density	0.210	0.209	0.208	0.151	0.166	0.163	0.347
	(0.011)***	(0.012)***	(0.012)***	(0.012)***	(0.011)***	(0.009)***	(0.086)***
Pop.size	0.013	0.070	0.079	0.093	0.017	− 0.057	− 0.345
	(0.011)	(0.012)***	(0.013)***	(0.011)***	(0.010)*	(0.011)***	(0.105)***
Elevation	0.027	0.029	0.007	0.105	0.058	0.020	0.085
	(0.011)**	(0.013)***	(0.013)	(0.010)***	(0.013)***	(0.018)	(0.164)
Sea prox.	0.097	0.130	0.126	0.081	0.062	0.073	0.209
	(0.010)***	(0.011)***	(0.011)***	(0.009)***	(0.008)***	(0.013)	(0.151)
Cap.city	0.060	0.040	0.039	0.059	0.064	0.065	0.033
	(0.010)***	(0.010)***	(0.010)***	(0.008)***	(0.007)***	(0.007)***	(0.051)
Lag. Pop.growth			− 0.137 (0.057)**				
Lag.Dem.density			− 0.225 (0.050)***				
Lag.Pop.size			− 0.210 (0.077)***				
Lag.Elevation			0.248 (0.039)***				
Lag.Sea prox.			(0.041)*** − 0.264				
Lag.Cap.city			0.916 (0.569)				
Breusch-Pagan							
Durbin-Watson							
Slope equality							
Moran’s I(z)				0.053***			
W spatial matrix				***	***	***	***
Adjusted-$R^2$	0.237	0.284	0.292				
AIC	20,532	20,147	20,231				

### From compact urbanisation to suburbanisation

Table 4 illustrates the results of econometric modeling investigating a distinctive time interval as far as population dynamics in Italy is concerned. Both OLS and quantile regressions improved their goodness-of-fit (adjusted-$R^2$ $> 0.4$), corresponding to contained values of AIC, at least in OLS and in the first three quartiles of standard quartile regressions. Lagged population growth rates and demographic density have a positive impact of current population growth rates; population size, elevation, proximity to the sea coast and head town have a negative–while less intense and more mixed–impact on current population growth rates. Following the results of econometric diagnostics, spatial models provide outcomes adjusted to the spatial structure of both predictors and the dependent variable. However, in the essence, global spatial models give results well aligned with those of the OLS regression; moreover, spatial quantile regressions confirm the outcomes of standard quantile regressions. Coherent results are also displayed comparing adjusted-$R^2$ with AIC, i.e., higher values of the first indicator correspond to lower values of the second one. Adjusted-$R^2$ confirms the best performance of SDM in both cases, while low performances (low adjusted-$R^2$ and high AIC), are found in the two specifications of SAR.

Results of both OLS and quantile regressions for the subsequent time interval (Table 5) are also aligned with the models’ outcomes described for the previous decade (see above), with model’s goodness-of-fit maintaining generally high. Lagged population growth rates, demographic density, and proximity to the sea coast reveal a positive and significant impact on the dependent variable. Elevation and head town show the reverse effect, while population size display more mixed results, possible associated with a higher spatial heterogeneity characteristic of this predictor. Although the non-redundant structure of predictors (VIF systematically below 2), econometric diagnostics suggested the appropriateness of spatial modelling also in this case. Improving slightly the goodness-of-fit in respect with non-spatial modeling, results of global regressions (SAR, SDE and SDM, direct effects) are mostly aligned with the OLS model. Moreover, spatial quantile regressions estimate a comparable structure of coefficients for the selected predictors, irrespective of the spatial weighting scheme. All these models show appreciable goodness-of-fit that reflects convergent results, as far as sign and significance of regression coefficients. Based on the values of AIC, the best performance is attributed to SDE and SDM weighted by distance In Table 5. Conversely, SAR model exhibits a lower value of AIC with contiguity. All outcomes are in line with adjusted-$R^2$.

### Moving toward counter-urbanisation

Similarly to what reported in Tables 4 and 5, modeling population growth rates between 1981 and 1991 (Table 6) with standard econometrics delineate the role of head town (negative impact) as well as lagged population growth rates and demographic density (positive impacts). In partial disagreement with what has been observed in earlier decades, population size, elevation, and proximity to the sea coast, show more heterogeneous results, with significant and non-significant coefficients and contrasting signs when moving from OLS to quantile regressions. Despite a satisfactory goodness-of-fit, econometric diagnostics suggested the use of spatial models that reached a systematically higher fit than standard models. Results of global models, irrespective of the spatial weighting scheme, confirm the role of lagged population growth rates and demographic density as predictors of current population growth rates. Outcomes from AIC, confirmed by adjusted-$R^2$, follow the same tendency. Elevation and population size give more mixed, while significant, results. Quantile regressions provide useful insights when describing the latent heterogeneity associated with predictors such as population size, elevation, proximity to the sea coast and head town. For instance, the negative impact of population size is spatially polarized, i.e. higher for the forth quartile and lower moving from the third to the first quartile. A reverse pattern is observed for elevation and proximity to the sea coast.

**Table 4:** Results of standard (OLS, Ordinary Least Square, and quantile) regressions as well as global (SAR: Spatial Autoregressive model; SDE: Spatial Error model; SDM: Spatial Durbin model) and quantile spatial models run with both contiguity and linear distance spatial weighting matrices; population growth rate (% annual) in 1961–1971 as dependent variable; population growth rate (1951–1961), demographic density (1951), population size (1951), elevation, proximity to the sea coast and a dummy indicating municipalities that act as provincial head town as predictors (*$p< 0.05$; **$0.001<$ $p< 0.05$; ***$p< 0.001$).

Predictor	OLS	Quantile regression				VIF	
		$\tau = 0.25$	$\tau = 0.50$	$\tau = 0.75$	$\tau = 0.99$		
Intercept	$\approx 0.000$	− 0.329	− 0.051	0.235	1.966		
	(0.008)	(0.006)***	(0.005)***	(0.007)***	(0.162)***		
Pop.growth	0.547	0.499	0.567	0.646	1.363	1.13	
	(0.009)***	(0.008)***	(0.008)***	(0.010)***	(0.179)***		
Dem.density	0.188	0.146	0.157	0.132	0.244	1.56	
	(0.010)***	(0.007)***	(0.007)***	(0.008)***	(0.161)		
Pop.size	− 0.029	0.039	− 0.011	− 0.048	− 0.496	1.53	
	(0.010)***	(0.006)***	(0.006)*	(0.007)***	(0.116)***		
Elevation	− 0.071	− 0.08	− 0.073	− 0.090	− 0.055	1.46	
	(0.010)***	(0.004)***	(0.005)***	(0.007)***	(0.109)		
Sea prox.	− 0.035	− 0.033	− 0.034	− 0.047	0.054	1.20	
	(0.009)***	(0.005)***	(0.004)***	(0.007)***	(0.178)		
Cap.city	− 0.015	− 0.011	− 0.011	− 0.017	− 0.048	1.14	
	(0.009)*	(0.006)*	(0.003)***	(0.005)***	(0.144)		
Lag. Pop.growth							
Lag.Dem.density							
Lag.Pop.size							
Lag.Elevation							
Lag.Sea prox.							
Lag.Cap.city							
Breusch-Pagan	238.2***						
Durbin-Watson	1.83***						
Slope equality			48.1***				
Moran’s I(z)							
W spatial matrix							
Adjusted-$R^2$	0.403	0.327	0.361	0.379	0.353		
AIC	18,821	12,164	11,953	14,735	37,300		

Predictor	Contiguity-based spatial weights						
	SAR	SDE	SDM	Quantile regression			
				$\tau = 0.25$	$\tau = 0.50$	$\tau = 0.75$	$\tau = 0.99$
Intercept	$\approx 0.000$	− 0.001	− 0.001	− 0.310	− 0.049	0.230	1.895
	(0.008)	(0.009)	(0.008)	(0.006)***	(0.005)***	(0.007)***	(0.237)***
Pop.growth	0.539	0.543	0.534	0.492	0.563	0.641	1.322
	(0.009)***	(0.009)***	(0.009)***	(0.013)***	(0.011)***	(0.013)***	(0.123)***
Dem.density	0.178	0.183	0.171	0.140	0.154	0.130	0.218
	(0.010)***	(0.010)***	(0.011)***	(0.008)***	(0.008)***	(0.008)***	(0.128)*
Pop.size	− 0.020	− 0.016	0.012	0.041	− 0.008	− 0.046	− 0.489
	(0.010)*	(0.010)	(0.011)	(0.006)***	(0.006)	(0.007)***	(0.113)***
Elevation	− 0.067	− 0.065	− 0.049	− 0.074	− 0.07	− 0.089	− 0.115
	(0.010)***	(0.010)***	(0.011)***	(0.006)***	(0.006)***	(0.007)***	(0.090)
Sea prox.	− 0.029	− 0.023	0.002	− 0.031	− 0.031	− 0.045	0.072
	(0.009)***	(0.009)**	(0.010)	(0.006)***	(0.006)***	(0.006)***	(0.119)
Cap.city	− 0.015	− 0.018	− 0.021	− 0.013	− 0.012	− 0.014	− 0.033
	(0.009)*	(0.009)**	(0.009)**	(0.007)*	(0.003)***	(0.005)***	(0.033)
Lag. Pop.growth			− 0.021				
			(0.020)				
Lag.Dem.density			0.022				
			(0.020)				
Lag.Pop.size			− 0.119				
			(0.019)***				
Lag.Elevation			0.047				
			(0.018)**				
Lag.Sea prox.			− 0.106				
			(0.017)***				
Lag.Cap.city			0.046				
			(0.020)**				
Breusch-Pagan							
Durbin-Watson							
Slope equality							
Moran’s I(z)				0.099***			
W spatial matrix				***	***	*	ns
Adjusted-$R^2$	0.408	0.409	0.416				
AIC	33,100	33,015	33,011				

Predictor	Distance-based spatial weights						
	SAR	SDE	SDM	Quantile regression			
				$\tau = 0.25$	$\tau = 0.50$	$\tau = 0.75$	$\tau = 0.99$
Intercept	0.003	0.044	0.002	− 0.295	− 0.053	0.209	1.951
	(0.008)	(0.108)	(0.008)	(0.006)***	(0.005)***	(0.007)***	(0.214)***
Pop.growth	0.525	0.531	0.526	0.479	0.540	0.621	1.318
	(0.009)***	(0.009)***	(0.009)***	(0.014)***	(0.009)***	(0.010)***	(0.155)***
Dem.density	0.172	0.175	0.175	0.131	0.142	0.131	0.240
	(0.010)***	(0.011)***	(0.011)***	(0.007)***	(0.008)***	(0.009)***	(0.142)*
Pop.size	0.028	0.050	0.054	0.085	0.036	− 0.010	− 0.481
	(0.010)***	(0.011)***	(0.012)***	(0.007)***	(0.008)***	(0.008)	(0.127)***
Elevation	− 0.042	− 0.029	− 0.036	− 0.043	− 0.049	− 0.006	− 0.084
	(0.010)***	(0.011)**	(0.012)***	(0.006)***	(0.007)***	(0.008)	(0.117)
Sea prox.	0.002	0.028	0.027	− 0.006	− 0.001	− 0.025	0.109
	(0.009)	(0.010)***	(0.010)***	(0.005)	(0.005)	(0.006)***	(0.123)
Cap.city	− 0.025	− 0.035	− 0.035	− 0.018	− 0.021	− 0.015	− 0.038
	(0.009)***	(0.009)***	(0.009)***	(0.006)***	(0.003)***	(0.006)	(0.035)
Lag. Pop.growth			− 0.331				
			(0.065)***				
Lag.Dem.density			− 0.137				
			(0.038)***				
Lag.Pop.size			− 0.154				
			(0.061)**				
Lag.Elevation			0.081				
			(0.035)**				
Lag.Sea prox.			− 0.115				
			(0.427)***				
Lag.Cap.city			0.742				
			(0.414)*				
Breusch-Pagan							
Durbin-Watson							
Slope equality							
Moran’s I(z)				0.039***			
W spatial matrix				***	***	***	ns
Adjusted-$R^2$	0.418	0.429	0.430				
AIC	32,948	32,741	32,796

**Table 5:** Results of standard (OLS, Ordinary Least Square, and quantile) regressions as well as global (SAR: Spatial Autoregressive model; SDE: Spatial Error model; SDM: Spatial Durbin model) and quantile spatial models run with both contiguity and linear distance spatial weighting matrices; population growth rate (% annual) in 1971–1981 as dependent variable; population growth rate (1961–1971), demographic density (1961), population size (1961), elevation, proximity to the sea coast and a dummy indicating municipalities that act as provincial head town as predictors (*$p< 0.05$; **$0.001<$ $p< 0.05$; ***$p< 0.001$).

Predictor	OLS	Quantile regression				VIF	
		$\tau = 0.25$	$\tau = 0.50$	$\tau = 0.75$	$\tau = 0.99$		
Intercept	$\approx 0.000$	− 0.424	− 0.074	0.324	2.358		
	(0.009)	(0.007)***	(0.007)***	(0.010)***	(0.131)***		
Pop.growth	0.0452	0.502	0.610	0.727	1.361	1.24	
	(0.010)***	(0.014)***	(0.014)	(0.018)***	(0.194)***		
Dem.density	0.123	0.038	0.048	0.077	0.281	1.82	
	(0.012)***	(0.010)***	(0.010)***	(0.013)***	(0.136)***		
Pop.size	0.008	0.128	0.077	− 0.029	− 0.735	1.65	
	(0.011)	(0.008)***	(0.008)***	(0.010)***	(0.094)***		
Elevation	− 0.115	− 0.089	− 0.069	− 0.078	− 0.253	1.45	
	(0.010)***	(0.007)***	(0.008)***	(0.010)***	(0.148)*		
Sea prox.	0.040	0.003	0.015	0.035	0.074	1.15	
	(0.009)***	(0.007)	(0.007)**	(0.010)***	(0.113)		
Cap.city	− 0.066	− 0.083	− 0.082	− 0.055	$\approx 0.000$	1.14	
	(0.009)***	(0.011)***	(0.010)***	(0.008)***	(0.117)		
Lag. Pop.growth							
Lag.Dem.density							
Lag.Pop.size							
Lag.Elevation							
Lag.Sea prox.							
Lag.Cap.city							
Breusch-Pagan	2703.5***						
Durbin-Watson	1.88***						
Slope equality			52.7***				
Moran’s I(z)							
W spatial matrix							
Adjusted-$R^2$	0.326	0.258	0.254	0.245	0.309		
AIC	19,799	16,600	16,412	19,228	37,343		

Predictor	Contiguity-based spatial weights						
	SAR	SDE	SDM	Quantile regression			
				$\tau = 0.25$	$\tau = 0.50$	$\tau = 0.75$	$\tau = 0.99$
Intercept	$\approx 0.000$	$\approx 0.000$	$\approx 0.000$	− 0.418	− 0.074	0.319	2.146
	(0.009)	(0.009)	(0.009)	(0.008)***	(0.007)***	(0.011)***	(0.169)***
Pop.growth	0.045	0.451	0.451	0.502	0.605	0.727	1.366
	(0.010)***	(0.010)***	(0.010)***	(0.023)***	(0.024)***	(0.253)***	(0.115)***
Dem.density	0.119	0.122	0.106	0.035	0.047	0.072	0.279
	(0.012)***	(0.012)***	(0.013)***	(0.012)***	(0.012)***	(0.011)***	(0.118)**
Pop.size	0.008	0.005	$\approx 0.000$	0.129	0.076	− 0.031	− 0.765
	(0.011)	(0.011)	(0.012)	(0.007)***	(0.008)***	(0.011)*	(0.066)***
Elevation	− 0.114	− 0.118	− 0.142	− 0.088	− 0.069	− 0.079	− 0.192
	(0.010)***	(0.011)***	(0.012)***	(0.007)***	(0.008)***	(0.011)***	(0.174)
Sea prox.	0.041	0.040	0.034	0.004	0.014	0.036	0.072
	(0.009)***	(0.009)***	(0.010)***	(0.007)	(0.007)*	(0.011)**	(0.149)
Cap.city	− 0.065	− 0.065	− 0.063	− 0.083	− 0.080	− 0.054	0.012
	(0.009)***	(0.009)***	(0.009)***	(0.009)***	(0.009)***	(0.009)***	(0.028)
Lag. Pop.growth			0.028				
			(0.021)				
Lag.Dem.density			0.039				
			(0.023)*				
Lag.Pop.size			0.106				
			(0.022)***				
Lag.Elevation			0.109				
			(0.019)***				
Lag.Sea prox.			− 0.026				
			(0.018)				
Lag.Cap.city			− 0.040				
			(0.022)*				
Breusch-Pagan							
Durbin-Watson							
Slope equality							
Moran’s I(z)				0.069***			
W spatial matrix				ns	ns	ns	ns
Adjusted-$R^2$	0.331	0.330	0.335				
AIC	19,784	19,763	19,761				

Predictor	Distance-based spatial weights						
	SAR	SDE	SDM	Quantile regression			
				$\tau = 0.25$	$\tau = 0.50$	$\tau = 0.75$	$\tau = 0.99$
Intercept	$\approx 0.000$	0.016	0.002	− 0.423	− 0.075	0.326	2.36023
	(0.009)	(0.037)	(0.009)	(0.007)***	(0.007)***	(0.012)***	(0.108)***
Pop.growth	0.452	0.461	0.455	0.501	0.609	0.734	1.361
	(0.010)***	(0.010)***	(0.010)***	(0.024)***	(0.023)***	(0.023)***	(0.107)***
Dem.density	0.123	0.153	0.163	0.036	0.049	0.073	0.249
	(0.012)***	(0.013)***	(0.013)***	(0.012)***	(0.012)***	(0.011)***	(0.098)***
Pop.size	0.008	− 0.036	− 0.046	0.128	0.075	− 0.027	− 0.763
	(0.012)	(0.013)***	(0.013)***	(0.008)***	(0.008)***	(0.012)**	(0.071)***
Elevation	− 0.115	− 0.116	− 0.126	− 0.087	− 0.071	− 0.085	− 0.279
	(0.011)***	(0.012)***	(0.012)***	(0.008)***	(0.007)***	(0.011)***	(0.143)*
Sea prox.	0.040	0.040	0.038	0.006	0.013	0.031	0.066
	(0.010)***	(0.010)***	(0.010)***	(0.007)	(0.006)**	(0.011)***	(0.145)
Cap.city	− 0.066	− 0.059	− 0.060	− 0.083	− 0.082	− 0.055	− 0.002
	(0.009)***	(0.009)***	(0.009)***	(0.008)***	(0.008)***	(0.009)***	(0.027)
Lag. Pop.growth			0.453				
			(0.140)***				
Lag.Dem.density			− 0.518				
			(0.060)***				
Lag.Pop.size			0.743				
			(0.102)***				
Lag.Elevation			0.227				
			(0.046)***				
Lag.Sea prox.			− 0.244				
			(0.041)***				
Lag.Cap.city			− 1.588				
			(0.454)***				
Breusch-Pagan							
Durbin-Watson							
Slope equality							
Moran’s I(z)				0.008***			
W spatial matrix				ns	ns	ns	ns
Adjusted-$R^2$	0.329	0.335	0.340				
AIC	19,801	19,676	19,676

**Table 6:** Results of standard (OLS, Ordinary Least Square, and quantile) regressions as well as global (SAR: Spatial Autoregressive model; SDE: Spatial Error model; SDM: Spatial Durbin model) and quantile spatial models run with both contiguity and linear distance spatial weighting matrices; population growth rate (% annual) in 1981–1991 as dependent variable; population growth rate (1971–1981), demographic density (1971), population size (1971), elevation, proximity to the sea coast and a dummy indicating municipalities that act as provincial head town as predictors (*$p< 0.05$; **$0.001<$ $p< 0.05$; ***$p< 0.001$).

Predictor	OLS	Quantile regression				VIF	
		$\tau = 0.25$	$\tau = 0.50$	$\tau = 0.75$	$\tau = 0.99$		
Intercept	$\approx 0.000$	− 0.344	− 0.043	0.280	1.819		
	(0.009)	(0.007)***	(0.006)***	(0.009)***	(0.120)***		
Pop.growth	0.528	0.459	0.521	0.604	1.092	1.27	
	(0.010)***	(0.009)***	(0.009)***	(0.012)***	(0.134)***		
Dem.density	0.084	0.041	0.065	0.092	0.281	1.94	
	(0.012)***	(0.008)***	(0.007)***	(0.008)***	(0.109)**		
Pop.size	− 0.078	0.047	− 0.038	− 0.135	− 0.566	1.87	
	(0.012)***	(0.007)***	(0.007)***	(0.007)***	(0.080)***		
Elevation	− 0.027	− 0.014	0.020	0.028	0.179	1.43	
	(0.011)*	(0.005)***	(0.005)***	(0.007)***	(0.058)***		
Sea prox.	0.010	− 0.006	0.019	0.035	0.114	1.19	
	(0.010)	(0.006)	(0.005)***	(0.008)***	(0.043)***		
Cap.city	− 0.029	− 0.051	− 0.027	− 0.019	0.062	1.15	
	(0.009)**	(0.009)***	(0.005)***	(0.003)***	(0.189)		
Lag. Pop.growth							
Lag.Dem.density							
Lag.Pop.size							
Lag.Elevation							
Lag.Sea prox.							
Lag.Cap.city							
Breusch-Pagan	376.9***						
Durbin-Watson	1.94**						
Slope equality			95.1***				
Moran’s I(z)							
W spatial matrix							
Adjusted-$R^2$	0.309	0.260	0.254	0.256	0.295		
AIC	20,006	14,525	13,873	16,413	35,423		

Predictor	Contiguity-based spatial weights						
	SAR	SDE	SDM	Quantile regression			
				$\tau = 0.25$	$\tau = 0.50$	$\tau = 0.75$	$\tau = 0.99$
Intercept	$\approx 0.000$	$\approx 0.000$	$\approx 0.000$	− 0.325	− 0.042	0.266	1.600
	(0.009)	(0.009)	(0.009)	(0.008)***	(0.006)***	(0.010)***	(0.173)***
Pop.growth	0.526	0.527	0.523	0.459	0.519	0.597	1.093
	(0.010)***	(0.010)***	(0.010)***	(0.015)***	(0.014)***	(0.019)***	(0.113)***
Dem.density	0.078	0.082	0.064	0.030	0.062	0.084	0.231
	(0.012)***	(0.013)***	(0.013)***	(0.011)**	(0.010)***	(0.011)***	(0.121)*
Pop.size	− 0.078	− 0.080	− 0.080	0.049	− 0.039	− 0.138	− 0.537
	(0.012)***	(0.012)***	(0.013)***	(0.010)***	(0.008)***	(0.011)***	(0.090)***
Elevation	− 0.027	− 0.030	− 0.052	− 0.016	0.020	0.026	0.127
	(0.011)**	(0.011)**	(0.012)***	(0.005)***	(0.006)***	(0.005)***	(0.102)
Sea prox.	0.011	0.010	0.022	− 0.006	0.021	0.037	0.094
	(0.010)	(0.010)	(0.010)	(0.005)	(0.005)***	(0.009)***	(0.073)
Cap.city	− 0.029	− 0.029	− 0.027	− 0.052	− 0.028	− 0.018	0.038
	(0.009)***	(0.009)***	(0.009)***	(0.007)***	(0.005)***	(0.003)***	(0.043)
Lag. Pop.growth			0.022				
			(0.024)				
Lag.Dem.density			0.055				
			(0.024)**				
Lag.Pop.size			0.051				
			(0.024)**				
Lag.Elevation			0.090				
			(0.020)***				
Lag.Sea prox.			0.011				
			(0.018)				
Lag.Cap.city			− 0.024				
			(0.022)				
Breusch-Pagan							
Durbin-Watson							
Slope equality							
Moran’s I(z)				0.057***			
W spatial matrix				***	***	***	*
Adjusted-$R^2$	0.311	0.310	0.313				
AIC	19,988	19,976	19,976				

Predictor	Distance-based spatial weights						
	SAR	SDE	SDM	Quantile regression			
				$\tau = 0.25$	$\tau = 0.50$	$\tau = 0.75$	$\tau = 0.99$
Intercept	$\approx 0.000$	− 0.004	− 0.006	− 0.337	− 0.043	0.278	1.859
	(0.009)	(0.035)	(0.009)	(0.007)***	(0.006)***	(0.010)***	(0.122)***
Pop.growth	0.528	0.530	0.526	0.459	0.522	0.604	1.052
	(0.010)***	(0.010)***	(0.010)***	(0.013)***	(0.013)***	(0.017)***	(0.127)***
Dem.density	0.083	0.090	0.088	0.036	0.064	0.092	0.270
	(0.012)***	(0.014)***	(0.014)***	(0.010)***	(0.009)***	(0.009)***	(0.101)***
Pop.size	− 0.078	− 0.103	− 0.106	0.046	− 0.038	− 0.137	− 0.508
	(0.012)***	(0.013)***	(0.014)***	(0.008)***	(0.009)***	(0.011)***	(0.101)***
Elevation	− 0.026	− 0.055	− 0.072	− 0.012	0.019	0.028	0.198
	(0.011)**	(0.012)***	(0.013)***	(0.005)**	(0.006)***	(0.008)***	(0.115)*
Sea prox.	0.012	0.001	− 0.003	− 0.002	0.020	0.036	0.124
	(0.010)	(0.011)	(0.011)	(0.006)	(0.005)***	(0.008)***	(0.072)*
Cap.city	− 0.026	− 0.025	− 0.026	− 0.015	− 0.027	− 0.019	0.020
	(0.009)***	(0.009)**	(0.009)**	(0.007)***	(0.004)***	(0.004)***	(0.053)
Lag. Pop.growth			0.123				
			(0.150)				
Lag.Dem.density			− 0.126				
			(0.050)**				
Lag.Pop.size			0.442				
			(0.87)***				
Lag.Elevation			0.335				
			(0.051)***				
Lag.Sea prox.			− 0.189				
			(0.045)***				
Lag.Cap.city			− 0.839				
			(0.439)*				
Breusch-Pagan							
Durbin-Watson							
Slope equality							
Moran’s I(z)				0.003***			
W spatial matrix				**	ns	ns	ns
Adjusted-$R^2$	0.309	0.313	0.3416				
AIC	20,008	19,926	19,928				

Standard and spatial modeling explaining the variability in population growth rates (1991–2001) gain significance, with a considerable goodness-of-fit increasing further when the spatial structure of predictors is considered (Table 7). Lagged population growth rates and demographic density show a positive and highly significant coefficient irrespective of the model used. The impact of population size (negative and significant coefficients) is also homogeneous across model’s specifications. The impact of elevation is also negative, although minor differences were found comparing the results of quantile regressions run with the two spatial weighting schemes. Global models (both standard and spatial) also suggest how proximity to the sea coast exerted a negative and significant impact on the dependent variable. Quantile regressions provide similar results, with the only exception of the forth quartile. Head town as a predictor of population growth rates is associated to mostly negative coefficients (global models). Outcomes of adjusted-$R^2$ and AIC, jointly confirm the better performance of distance weighted models compared to contiguity. Negative coefficients were also found in quantile regressions (both standard and spatial) for the first and the second quartiles, but not for the third and the forth quartiles.

### Latent trends toward re-urbanisation

Table 8 shows the results of econometric models estimating the spatial variability of population growth rates between 2001 and 2011. All models, starting from OLS, significantly improved their goodness-of-fit compared with what has been estimated in earlier decades. Econometric diagnostics, goodness-of-fit (adjusted-$R^2$ or pseudo-$R^2$) and the Akaike Information Criterion (AIC) coherently document how spatial models performed better than standard models. Concerning spatial models, in line with adjusted-$R^2$, the distance weighted scenario seems to better perform than contiguity. Lagged population growth rates and demographic density have a positive and highly significant impact on the dependent variable, irrespective of the model’s specification and the spatial weighting scheme. Global and quantile models provide comparable results for these two predictors. The impact of elevation is, in turn, negative and highly significant, for all models. However, the indirect impact of this variable estimated via SDM results slightly significant or completely insignificant. The impact of the proximity to the sea coast and head town as predictors of population growth rates is negative - as depicted in the outcomes of global (both standard and spatial) econometric models - and more mixed in quantile regressions, being moderately significant for the first and second quartiles (proximity to the sea coast) and for the first and forth quartiles (head town).

The outcomes of regressions modeling the spatial variability of population growth rates between 2011 and 2021 indicate substantially different demographic dynamics, as far as intensity and spatial structure are concerned (Table 9). The overall estimate of model’s goodness-of-fit is moderate or rather low for all econometric specifications, and improved slightly moving from standard to spatial models, despite test diagnostics were convergent in suggesting the appropriateness of using spatially explicit approaches. From the results of AIC and adjusted-$R^2$ in spatially weighted models, it emerges the higher fit of distance models in comparison with contiguity models. Quantile regressions performed better than global models; more specifically, results for the first and second quartiles had a satisfactory goodness-of-fit, declining for the third and forth quartiles. Among predictors, the impact of lagged population growth rates on the dependent variable is confirmed, in line with the results of estimates for previous decades, although (global) regression coefficients are less intense and significant, irrespective of the model’s specification. The same (positive) impact was found in quantile regressions (first to third quartile), with the exception of the forth quartile. The impact of demographic density is really mixed and heterogeneous moving from global to quantile regressions. Population size seems to have a slightly negative impact on the dependent variable, although econometric estimates were rather mixed, as in the case of demographic density. Elevation, proximity to the sea coast and head town display almost insignificant and close-to-zero regression coefficients, apart from few exceptions (mainly in quantile regressions).

**Table 7:** Results of standard (OLS, Ordinary Least Square, and quantile) regressions as well as global (SAR: Spatial Autoregressive model; SDE: Spatial Error model; SDM: Spatial Durbin model) and quantile spatial models run with both contiguity and linear distance spatial weighting matrices; population growth rate (% annual) in 1991–2001 as dependent variable; population growth rate (1981–1991), demographic density (1981), population size (1981), elevation, proximity to the sea coast and a dummy indicating municipalities that act as provincial head town as predictors (*$p< 0.05$; ** $0.001<$ $p< 0.05$; *** $p< 0.001$).

Predictor	OLS	Quantile regression				VIF	
		$\tau = 0.25$	$\tau = 0.50$	$\tau = 0.75$	$\tau = 0.99$		
Intercept	$\approx 0.000$	− 0.431	− 0.030	0.402	2.069		
	(0.009)	(0.009)***	(0.008)***	(0.010)***	(0.100)***		
Pop.growth	0.455	0.601	0.718	0.822	0.969	1.13	
	(0.009)***	(0.016)***	(0.014)***	(0.017)***	(0.126)***		
Dem.density	0.204	0.102	0.099	0.114	0.181	2.04	
	(0.013)***	(0.012)***	(0.011)***	(0.011)***	(0.114)		
Pop.size	− 0.126	0.005	− 0.074	− 0.195	− 0.669	2.03	
	(0.013)***	(0.011)	(0.010)***	(0.010)***	(0.097)***		
Elevation	− 0.128	− 0.097	− 0.076	− 0.088	− 0.309	1.44	
	(0.011)***	(0.008)***	(0.008)***	(0.011)***	(0.098)***		
Sea prox.	− 0.103	− 0.105	− 0.095	− 0.090	− 0.053	1.18	
	(0.010)***	(0.009)***	(0.008)***	(0.009)***	(0.119)		
Cap.city	− 0.030	− 0.030	− 0.015	0.005	0.088	1.13	
	(0.009)**	(0.004)***	(0.004)***	(0.004)	(0.082)		
Lag. Pop.growth							
Lag.Dem.density							
Lag.Pop.size							
Lag.Elevation							
Lag.Sea prox.							
Lag.Cap.city							
Breusch-Pagan	6,326.6***						
Durbin-Watson	1.70***						
Slope equality			78.7***				
Moran’s I(z)							
W spatial matrix							
Adjusted-$R^2$	0.368	0.236	0.242	0.252	0.305		
AIC	21,793	20,470	19,487	21,374	35,767		

Predictor	Contiguity-based spatial weights						
	SAR	SDE	SDM	Quantile regression			
				$\tau = 0.25$	$\tau = 0.50$	$\tau = 0.75$	$\tau = 0.99$
Intercept	− 0.001	− 0.002	− 0.001	− 0.377	− 0.029	0.353	1.826
	(0.009)	(0.012)	(0.008)	(0.012)***	(0.008)***	(0.011)***	(0.168)***
Pop.growth	0.445	0.445	0.447	0.596	0.702	0.812	0.951
	(0.009)***	(0.009)***	(0.009)***	(0.022)***	(0.018)***	(0.020)***	(0.110)***
Dem.density	0.181	0.196	0.178	0.078	0.081	0.090	0.173
	(0.013)***	(0.013)***	(0.014)***	(0.014)***	(0.012)***	(0.014)***	(0.089)*
Pop.size	− 0.106	− 0.104	− 0.084	0.023	− 0.058	− 0.179	− 0.641
	(0.012)***	(0.013)***	(0.013)***	(0.011)**	(0.012)***	(0.012)***	(0.067)***
Elevation	− 0.109	− 0.106	− 0.080	− 0.072	− 0.061	− 0.089	− 0.274
	(0.010)***	(0.011)***	(0.012)***	(0.008)***	(0.008)***	(0.013)***	(0.124)**
Sea prox.	− 0.073	− 0.061	− 0.030	− 0.077	− 0.067	− 0.070	0.043
	(0.009)***	(0.010)***	(0.010)***	(0.009)***	(0.008)***	(0.012)***	(0.102)
Cap.city	− 0.032	− 0.036	− 0.037	− 0.031	− 0.019	0.003	0.065
	(0.009)***	(0.009)***	(0.009)***	(0.006)***	(0.005)***	(0.006)	(0.037)*
Lag. Pop.growth			0.009				
			(0.025)				
Lag.Dem.density			− 0.029				
			(0.024)				
Lag.Pop.size			− 0.058				
			(0.024)**				
Lag.Elevation			− 0.074				
			(0.019)***				
Lag.Sea prox.			− 0.212				
			(0.018)***				
Lag.Cap.city			0.046				
			(0.021)**				
Breusch-Pagan							
Durbin-Watson							
Slope equality							
Moran’s I(z)				0.153***			
W spatial matrix				***	***	***	ns
Adjusted-$R^2$	0.337	0.336	0.350				
AIC	21,544	21,406	21,383				

Predictor	Distance-based spatial weights						
	SAR	SDE	SDM	Quantile regression			
				$\tau = 0.25$	$\tau = 0.50$	$\tau = 0.75$	$\tau = 0.99$
Intercept	− 0.004	− 0.012	− 0.005	− 0.369	− 0.038	0.317	2.053
	(0.008)	(0.131)	(0.009)	(0.009)***	(0.006)***	(0.011)***	(0.121)***
Pop.growth	0.457	0.452	0.449	0.635	0.733	0.819	0.980
	(0.009)***	(0.009)***	(0.009)***	(0.024)***	(0.017)***	(0.025)***	(0.097)***
Dem.density	0.164	0.193	0.195	0.028	0.043	0.107	0.174
	(0.013)***	(0.013)***	(0.014)***	(0.013)**	(0.012)***	(0.016)***	(0.103)*
Pop.size	− 0.056	− 0.069	− 0.073	0.071	− 0.027	− 0.159	− 0.654
	(0.012)***	(0.014)***	(0.014)***	(0.011)***	(0.011)**	(0.013)***	(0.082)***
Elevation	− 0.046	− 0.042	− 0.047	− 0.025	− 0.011	− 0.015	− 0.292
	(0.010)***	(0.012)***	(0.012)***	(0.009)***	(0.008)	(0.015)	(0.126)**
Sea prox.	0.005	0.011	0.011	− 0.013	0.005	0.013	− 0.049
	(0.009)	(0.010)***	(0.010)	(0.010)	(0.009)	(0.014)	(0.111)
Cap.city	− 0.043	− 0.046	− 0.045	− 0.039	− 0.023	0.004	0.085
	(0.009)***	(0.009)***	(0.009)***	(0.005)***	(0.005)***	(0.008)	(0.042)**
Lag. Pop.growth			− 0.521				
			(0.168)****				
Lag.Dem.density			− 0.274				
			(0.058)***				
Lag.Pop.size			0.237				
			(0.082)**				
Lag.Elevation			0.113				
			(0.037)***				
Lag.Sea prox.			− 0.176				
			(0.047)***				
Lag.Cap.city			− 0.200				
			(0.397)				
Breusch-Pagan							
Durbin-Watson							
Slope equality							
Moran’s I(z)				0.094***			
W spatial matrix				***	***	***	ns
Adjusted-$R^2$	0.368	0.374	0.376				
AIC	21,097	20,969	21,008

**Table 8:** Results of standard (OLS, Ordinary Least Square, and quantile) regressions as well as global (SAR: Spatial Autoregressive model; SDE: Spatial Error model; SDM: Spatial Durbin model) and quantile spatial models run with both contiguity and linear distance spatial weighting matrices; population growth rate (% annual) in 2001–2011 as dependent variable; population growth rate (1991–2001), demographic density (1991), population size (1991), elevation, proximity to the sea coast and a dummy indicating municipalities that act as provincial head town as predictors (*$p< 0.05$; ** $0.001<$ $p< 0.05$; ***$p< 0.001$).

Predictor	OLS	Quantile regression				VIF	
		$\tau = 0.25$	$\tau = 0.50$	$\tau = 0.75$	$\tau = 0.99$		
Intercept	$\approx 0.000$	− 0.407	− 0.077	0.315	2.093		
	(0.008)	(0.008)***	(0.007)***	(0.010)***	(0.102)***		
Pop.growth	0.614	0.576	0.635	0.669	0.862	1.17	
	(0.008)***	(0.008)***	(0.008)***	(0.012)***	(0.101)***		
Dem.density	0.111	0.066	0.102	0.135	0.412	2.20	
	(0.011)***	(0.009)***	(0.009)***	(0.014)***	(0.101)***		
Pop.size	− 0.054	0.073	− 0.017	− 0.114	− 0.733	2.15	
	(0.011)***	(0.008)***	(0.009)*	(0.012)***	(0.097)***		
Elevation	− 0.125	− 0.086	− 0.084	− 0.128	− 0.162	1.45	
	(0.009)***	(0.007)***	(0.008)***	(0.012)***	(0.046)***		
Sea prox.	− 0.046	− 0.054	− 0.041	− 0.041	0.030	1.20	
	(0.008)***	(0.006)***	(0.007)***	(0.010)***	(0.055)		
Cap.city	− 0.001	− 0.010	− 0.001	0.014	0.133	1.12	
	(0.008)	(0.004)**	(0.005)	(0.007)*	(0.070)*		
Lag. Pop.growth							
Lag.Dem.density							
Lag.Pop.size							
Lag.Elevation							
Lag.Sea prox.							
Lag.Cap.city							
Breusch-Pagan	2262.1***						
Durbin-Watson	1.88***						
Slope equality			47.9***				
Moran’s I(z)							
W spatial matrix							
Adjusted-$R^2$	0.475	0.335	0.326	0.308	0.290		
AIC	24,803	22,731	22,607	25,025	40,706		

Predictor	Contiguity-based spatial weights						
	SAR	SDE	SDM	Quantile regression			
				$\tau = 0.25$	$\tau = 0.50$	$\tau = 0.75$	$\tau = 0.99$
Intercept	$\approx 0.000$	$\approx 0.000$	$\approx 0.000$	− 0.371	− 0.071	0.278	1.916
	(0.003)	(0.009)	(0.007)	(0.008)***	(0.007)***	(0.011)***	(0.155)***
Pop.growth	0.598	0.605	0.584	0.549	0.607	0.641	0.855
	(0.008)***	(0.008)***	(0.008)***	(0.014)***	(0.011)***	(0.016)***	(0.080)***
Dem.density	0.101	0.109	0.094	0.060	0.093	0.116	0.346
	(0.011)***	(0.012)***	(0.012)***	(0.011)***	(0.010)***	(0.016)***	(0.129)***
Pop.size	− 0.042	− 0.047	− 0.022	0.083	− 0.003	− 0.095	− 0.710
	(0.011)***	(0.011)***	(0.012)*	(0.009)***	(0.010)	(0.014)***	(0.100)***
Elevation	− 0.113	− 0.125	− 0.118	− 0.069	− 0.076	− 0.112	− 0.147
	(0.009)***	(0.010)***	(0.011)***	(0.009)***	(0.010)***	(0.011)***	(0.068)**
Sea prox.	− 0.028	− 0.037	− 0.009	− 0.035	− 0.022	− 0.016	0.085
	(0.008)***	(0.008)***	(0.009)	(0.007)***	(0.007)***	(0.009)*	(0.073)
Cap.city	− 0.004	− 0.004	− 0.008	− 0.016	− 0.005	0.009	0.118
	(0.008)	(0.008)	(0.008)	(0.006)**	(0.006)	(0.006)	(0.039)***
Lag. Pop.growth			0.076				
			(0.021)***				
Lag.Dem.density			− 0.003				
			(0.022)				
Lag.Pop.size			− 0.058				
			(0.022)***				
Lag.Elevation			0.023				
			(0.017)				
Lag.Sea prox.			− 0.088				
			(0.016)***				
Lag.Cap.city			0.034				
			(0.019)*				
Breusch-Pagan							
Durbin-Watson							
Slope equality							
Moran’s I(z)				0.163***			
W spatial matrix				***	***	***	ns
Adjusted-$R^2$	0.483	0.478	0.488				
AIC	17,681	17,604	17,600				

Predictor	Distance-based spatial weights						
				Quantile regression			
	SAR	SDE	SDM	$\tau = 0.25$	$\tau = 0.50$	$\tau = 0.75$	$\tau = 0.99$
Intercept	− 0.002	− 0.023	0.005	− 0.393	− 0.083	0.288	2.110
	(0.007)	(0.067)	(0.008)	(0.007)***	(0.006)***	(0.010)***	(0.095)***
Pop.growth	0.58	0.574	0.571	0.520	0.584	0.618	0.862
	(0.008)***	(0.008)***	(0.008)***	(0.015)***	(0.010)***	(0.012)***	(0.080)***
Dem.density	0.102	0.105	0.110	0.060	0.089	0.141	0.390
	(0.011)***	(0.013)***	(0.013)***	(0.012)***	(0.011)***	(0.012)***	(0.131)**
Pop.size	− 0.020	− 0.020	− 0.023	0.113	0.013	− 0.095	− 0.718
	(0.011)***	(0.012)	(0.013)*	(0.009)***	(0.010)	(0.012)***	(0.102)***
Elevation	− 0.087	− 0.108	− 0.121	− 0.040	− 0.055	− 0.085	− 0.176
	(0.010)***	(0.010)***	(0.011)***	(0.008)***	(0.010)***	(0.012)***	(0.080)**
Sea prox.	0.004	$\approx 0.000$	− 0.001	− 0.007	0.013	0.016	0.016
	(0.009)	(0.009)	(0.009)	(0.008)	(0.008)	(0.010)	(0.076)
Cap.city	− 0.011	− 0.014	− 0.014	− 0.029	− 0.009	0.006	0.128
	(0.008)	(0.010)***	(0.008)*	(0.005)***	(0.008)	(0.007)	(0.041)***
Lag. Pop.growth			0.529				
			(0.186)***				
Lag.Dem.density			− 0.204				
			(0.061)***				
Lag.Pop.size			0.006				
			(0.084)				
Lag.Elevation			0.281				
			(0.042)***				
Lag.Sea prox.			− 0.072				
			(0.050)				
Lag.Cap.city			0.716				
			(0.456)				
Breusch-Pagan							
Durbin-Watson							
Slope equality							
Moran’s I(z)				0.115***			
W spatial matrix				***	***	***	ns
Adjusted-$R^2$	0.489	0.492	0.494				
AIC	24,040	23,906	23,932

**Table 9:** Results of standard (OLS, Ordinary Least Square, and quantile) regressions as well as global (SAR: Spatial Autoregressive model; SDE: Spatial Error model; SDM: Spatial Durbin model) and quantile spatial models run with both contiguity and linear distance spatial weighting matrices; population growth rate (% annual) in 2011–2021 as dependent variable; population growth rate (2001–2011), demographic density (2001), population size (2001), elevation, proximity to the sea coast and a dummy indicating municipalities that act as provincial head town as predictors (*$p< 0.05$; ** $0.001<$ $p< 0.05$; ***$p< 0.001$).

Predictor	OLS	Quantile regression				VIF	
		$\tau = 0.25$	$\tau = 0.50$	$\tau = 0.75$	$\tau = 0.99$		
Intercept	$\approx 0.000$	− 0.135	− 0.052	0.041	1.398		
	(0.011)	(0.001)***	(0.001)***	(0.002)***	(0.149)***		
Pop.growth	0.108	0.111	0.121	0.130	0.027	1.21	
	(0.012)***	(0.002)***	(0.002)***	(0.002)***	(0.080)		
Dem.density	− 0.007	0.021	0.026	0.030	− 0.069	2.29	
	(0.016)	(0.002)***	(0.002)***	(0.002)***	(0.126)		
Pop.size	− 0.051	0.050	0.020	− 0.010	− 0.460	2.23	
	(0.016)**	(0.002)***	(0.002)***	(0.003)***	(0.148)***		
Elevation	− 0.009	− 0.003	0.001	0.006	− 0.035	1.48	
	(0.013)	(0.001)*	(0.001)	(0.002)***	(0.114)		
Sea prox.	0.006	− 0.002	0.002	0.007	− 0.020	1.20	
	(0.012)	(0.001)	(0.001)	(0.001)***	(0.090)		
Cap.city	0.023	− 0.002	0.003	0.009	0.155	1.11	
	(0.011)*	(0.000)**	(0.001)	(0.002)***	(0.092)*		
Lag. Pop.growth							
Lag.Dem.density							
Lag.Pop.size							
Lag.Elevation							
Lag.Sea prox.							
Lag.Cap.city							
Breusch-Pagan	144.9***						
Durbin-Watson	1.98						
Slope equality			136.5***				
Moran’s I(z)							
W spatial matrix							
Adjusted-$R^2$	0.011	0.226	0.198	0.129	0.083		
AIC	44,275	20,402	20,681	25,805	61,907		

Predictor	Contiguity-based spatial weights						
				Quantile regression			
	SAR	SDE	SDM	$\tau = 0.25$	$\tau = 0.50$	$\tau = 0.75$	$\tau = 0.99$
Intercept	$\approx 0.000$	$\approx 0.000$	$\approx 0.000$	− 0.120	− 0.046	0.039	1.394
	(0.011)	(0.011)	(0.011)	(0.003)***	(0.002)***	(0.002)***	(0.213)***
Pop.growth	0.107	0.107	0.096	0.108	0.117	0.123	0.025
	(0.012)***	(0.012)***	(0.012)***	(0.002)***	(0.002)***	(0.003)***	(0.066)
Dem.density	− 0.007	− 0.007	− 0.009	0.018	0.022	0.025	− 0.074
	(0.016)	(0.016)	(0.018)	(0.003)***	(0.002)***	(0.003)***	(0.052)
Pop.size	− 0.005	− 0.050	− 0.038	0.053	0.024	− 0.006	− 0.451
	(0.016)***	(0.016)***	(0.017)**	(0.002)***	(0.002)***	(0.003)*	(0.086)***
Elevation	− 0.009	− 0.009	$\approx 0.000$	$\approx 0.000$	0.002	0.007	− 0.027
	(0.013)	(0.013)	(0.015)	(0.001)	(0.001)	(0.002)***	(0.052)
Sea prox.	0.007	0.007	0.023	$\approx 0.000$	0.004	0.014	− 0.019
	(0.012)	(0.012)	(0.013)*	(0.001)	(0.001)***	(0.002)***	(0.030)
Cap.city	0.023	0.023	0.020	− 0.001	0.003	0.007	0.153
	(0.011)**	(0.011)**	(0.011)*	(0.001)	(0.001)***	(0.001)***	(0.033)***
Lag. Pop.growth			0.032				
			(0.024)				
Lag.Dem.density			− 0.012				
			(0.030)				
Lag.Pop.size			− 0.037				
			(0.031)				
Lag.Elevation			− 0.025				
			(0.024)				
Lag.Sea prox.			− 0.051				
			(0.023)**				
Lag.Cap.city			− 0.005				
			(0.026)				
Breusch-Pagan							
Durbin-Watson							
Slope equality							
Moran’s I(z)				0.016***			
W spatial matrix				***	***	***	ns
Adjusted-$R^2$	0.012	0.011	0.013				
AIC	44,274	44,273	44,272				

Predictor	Distance-based spatial weights						
	SAR	SDE	SDM	Quantile regression			
				$\tau = 0.25$	$\tau = 0.50$	$\tau = 0.75$	$\tau = 0.99$
Intercept	0.002	0.007	− 0.004	− 0.126	− 0.050	0.037	1.226
	(0.011)	(0.025)	(0.011)	(0.001)***	(0.001)***	(0.002)***	(0.145)***
Pop.growth	0.096	0.094	0.090	0.102	0.109	0.117	− 0.016
	(0.012)***	(0.012)***	(0.012)***	(0.003)***	(0.003)***	(0.003)***	(0.044)
Dem.density	− 0.014	− 0.017	− 0.021	0.017	0.020	0.023	− 0.070
	(0.016)	(0.018)	(0.018)	(0.002)***	(0.002)***	(0.002)***	(0.033)**
Pop.size	− 0.036	− 0.034	− 0.028	0.060	0.031	0.003	− 0.357
	(0.016)	(0.017)*	(0.018)	(0.002)***	(0.002)***	(0.002)	(0.079)***
Elevation	− 0.001	− 0.001	$\approx 0.000$	0.003	0.005	0.013	− 0.035
	(0.013)	(0.014)	(0.015)	(0.001)**	(0.001)***	(0.002)***	(0.036)
Sea prox.	0.021	0.023	0.030	0.005	0.009	0.020	0.021
	(0.012)*	(0.013)*	(0.013)**	(0.001)***	(0.002)***	(0.002)***	(0.020)
Cap.city	0.020	0.020	0.018	− 0.004	$\approx 0.000$	0.006	0.089
	(0.011)*	(0.011)*	(0.011)	(0.001)***	(0.001)	(0.002)***	(0.026)***
Lag. Pop.growth			0.184				
			(0.103)				
Lag.Dem.density			− 0.014				
			(0.090)				
Lag.Pop.size			0.039				
			(0.115)				
Lag.Elevation			0.019				
			(0.057)				
Lag.Sea prox.			− 0.016				
			(0.064)				
Lag.Cap.city			− 0.509				
			(0.598)				
Breusch-Pagan							
Durbin-Watson							
Slope equality							
Moran’s I(z)				0.007***			
W spatial matrix				***	***	***	***
Adjusted-$R^2$	0.013	0.013	0.014				
AIC	44,261	44,265	44,266				

## Discussion

The present study illustrates a diachronic analysis of demographic dynamics at the municipal scale in Italy, verifying density-dependence, path-dependence, agglomeration/scale impacts, and spatial effects over a complete (demographic-urban) cycle from urbanisation to re-urbanisation. The approach developed in this study was based on the comparison of different statistical models, both parametric (global econometrics) and non-parametric (quantile regressions). This approach allowed a precise identification of the factors underlying processes of demographic growth and decline, in turn consolidating complex urban-rural hierarchies in advanced economies^75^. While global models provide a gross assessment of the impact of various predictors of population growth rate at a sufficiently detailed spatial scale^76^, quantile regressions allow an even more accurate inspection of the trends characteristic of specific parts of the statistical distribution of the dependent variable^77^. These statistical loci correspond to specific demographic behaviours, which reflect diversified but internally homogeneous territorial contexts. Examples include demographically dynamic contexts with positive and sustained growth rates, and demographically shrinking contexts having systematically negative growth rates. While non-spatial models (both global and quantile) provided the baseline knowledge to a refined understanding of population dynamics and the underlying factors and contexts, spatial models proved to be innovative tools analysing regional variability in the dependent variable. Moreover, the comparison between the results of global and quantile models specifying the geographical structure of the elementary units adopted in this study allows a more accurate examination of the role of spatial heterogeneity in population dynamics^78^. As far as the case study, while global models have satisfactorily explained population growth rates at a sufficiently detailed spatial level, the outcomes of quantile models often were in line with the results of global models. These outcomes were characteristic of the ‘urbanisation’ phase (1951–1961, 1961–1971, 1971–1981) with medium-high growth rates in Italy^79^. On the contrary, during the ’counter-urbanisation’ and ’re-urbanisation’ waves (1991–2001, 2001–2011, 2011–2021), global models fitted the dependent variable less effectively. At such times, quantile regressions provided likely more accurate indications of demographic behaviours in conditions of spatial heterogeneity^80^. This may highlight latent spatial patterns that are characteristic of territories with systematically high or low population growth rates^64^, in turn corresponding with specific quartiles of the statistical distribution of the dependent variable. Taken together, the results of the econometric models document how density-dependence has been observed in correspondence with urbanisation, suggesting a role for economic agglomeration and immigration^24^. Density-dependence was less significant over both suburbanisation and counter-urbanisation, when population tends to be more dispersed across regions^44^. In these contexts, the role of agglomeration and scale reduced proportionally, and path-dependent factors regulating population growth took the lead. With re-urbanisation, the positive rate of population growth observed in rural areas counterbalanced the stable (or negative) pattern observed in urban areas, indicating a relationship with population growth that reflects congestion externalities and subtle processes of peri-urbanisation intensifying in recent decades^5^. The outcomes of quantile regressions have more specifically delineated the existence of a non-linear relationship between population growth and density for all time intervals, although with important differences as far as the impact of individual factors is concerned. Results of quantile regressions document a positive effect of density on population growth rates, being stronger at higher levels of urban concentration, while declining slightly over time. Such findings are in line with the documented outcomes of sequential waves of urbanization, suburbanization and re-urbanization typical of post-war Italy^11,49,53^. In other words, the density-growth relationship is indicative of sequential stages characteristic of the metropolitan cycle in Mediterranean Europe^73^. All in all, our study demonstrates how sequential waves of concentration and de-concentration of urban and rural locations were associated with density-dependent mechanisms of population growth and decline. This process, shaping the expansion of rural/accessible districts, and the abandonment of marginal districts, accentuated the divide in high-density and low-density areas^63,81,82^. The flexibility of this approach justifies an extended use of global econometric models and quantile regressions analysing the drivers of population growth in socioeconomic contexts distinct from the one studied in this application, and at vastly differentiated (spatial and temporal) scales. An extensive use of spatial panel techniques applied to both global and quantile models is also recommended when sufficiently long time series of predictors are available at homogeneous and stable spatial units^77^. In addition, comparing the results of econometric models specifying multiple spatial weighting schemes (e.g. based on different contiguity and distance metrics) seems to be an appropriate tool^83^ when inferring about the stability of regression coefficients (sign and significance) across models^84^ and when taking decisions about the best performing models^85^. In this last case, diagnostics such as Akaike Information Criterion can easily and effectively complement such approaches^86^. Future studies should also investigate the appropriateness of local techniques (e.g. the Geographically Weighted Regression, GWR) to the investigation of regional variability and local heterogeneity in population dynamics^87^, considering both cross-section approaches and panel extensions, when input data allow such improvements. Although the process of demographic growth and decline at a local scale seem to follow similar underlying logics in various regions of Europe (for instance, in the Mediterranean countries^88^), a more accurate examination of the latent factors at the base of formation (and/or consolidation) of regional disparities in the geographical distribution and density of resident population may support a spatial planning aimed at a balanced, polycentric, and sustainable development of territories and local communities^89^. While identifying distinctive (demographic) regimes at the local scale, the empirical results of our study outline the intrinsic characteristics of local contexts and the differences in the relationship between population growth and density over time^15^, corroborating the assumption that density-dependent regulation was intrinsically associated with exogenous dynamics depending on urban cycles. In this perspective, long-term demographic processes in Mediterranean Europe^59^ can be seen as representative of more general dynamics at the continental scale. Based on a comparative approach^76^, our study definitely offers an exploratory econometric perspective to regional studies of population dynamics that can be easily adapted to different spatial scales (from local to regional levels), temporal schedule (from decadal to annual windows), and variables, e.g. moving from strictly demographic indicators to economic predictors of leading and lagging contexts in the old continent and beyond.

## Conclusions

A spatial econometric investigation of density-dependent and path-dependent mechanisms of population dynamics provided an original explanation of metropolitan cycles, delineating the evolution of socioeconomic (local) systems along the urban-rural gradient. With accelerated population dynamics, empirical results delineate compact urbanisation (1951–1981) as the main factor consolidating spatial disparities in Italy. As a matter of fact, econometric models–being only weakly significant in the inter-war period (1921–1951) - showed a high goodness-of-fit in correspondence with compact urbanisation that declined moderately with suburbanisation and counter-urbanisation (1981–2021). Density-dependence and path-dependence were found significant and, respectively, positive and negative, with compact urbanisation, and much less intense with suburbanisation and counter-urbanisation. The results of our study justify a renewed (diachronic and spatially explicit) analysis of socioeconomic development vis à vis demographic transition processes aimed at providing a more comprehensive interpretation of metropolitan transformations and the related evolution of local contexts.

## Data Availability

The datasets used and/or analysed during the current study available from the corresponding author on reasonable request.
